# A discrete choice experiment to assess people living with HIV's (PLWHIV's) preferences for GP or HIV clinic appointments

**DOI:** 10.1136/sextrans-2016-052643

**Published:** 2016-08-17

**Authors:** A H Miners, C D Llewellyn, V L Cooper, E Youssef, A J Pollard, M Lagarde, C Sabin, E Nixon, M Sachikonye, N Perry, M Fisher

**Affiliations:** 1 Faculty of Public Health and Policy, London School of Hygiene & Tropical Medicine, London, UK; 2 Division of Public Health and Primary Care, Brighton and Sussex Medical School, Brighton, UK; 3 Department of Genitourinary Medicine, Brighton and Sussex University Hospitals NHS Trust, Brighton, UK; 4 HIV Epidemiology & Biostatistics Group, University College London, London, UK; 5 UK Community Advisory Board Country (UKCAB), London, UK; 6 Brighton and Sussex Clinical Trials Unit, Brighton and Sussex Medical Schools, Brighton, UK

**Keywords:** HIV, HEALTH SERV RESEARCH, PRIMARY CARE, PATIENTS- VIEWS

## Abstract

**Objectives:**

To understand which aspects of general practitioner (GP) and HIV clinic appointments people living with HIV (PLWHIV) most value when seeking advice for new health problems.

**Methods:**

A discrete choice experiment using a convenience sample of people diagnosed with HIV. Participants were recruited from 14 general HIV clinics in the South East of England between December 2014 and April 2015. ORs were calculated using conditional logit (CLOGIT) and latent class models (LCMs).

**Results:**

A total of 1106 questionnaires were returned. Most participants were male (85%), white (74%) and were men who have sex with men (69%). The CLOGIT analysis showed people particularly valued shorter appointment waiting times (ORs between 1.52 and 3.62, p<0.001 in all instances). The LCM analysis showed there were two distinct classes, with 59% and 41% of respondents likely to be in each. The first class generally preferred GP to HIV clinic appointments and particularly valued ‘being seen quickly’. For example, they had strong preferences for shorter appointment waiting times and longer GP opening hours. People in the second class also valued shorter waiting times, but they had a strong general preference for HIV clinic rather than GP appointments.

**Conclusions:**

PLWHIV value many aspects of care for new health problems, particularly short appointment waiting times. However, they appear split in their general willingness to engage with GPs.

## Introduction

People living with HIV (PLWHIV) in resource-rich parts of the world who are promptly diagnosed and treated appropriately are now predicted to have similar life expectancies to uninfected people.^1^ As a consequence, the complexities of the medical needs of this group are changing, with a move away from the treatment of opportunistic infections towards the prevention and management of comorbidities associated with ageing such as cardiovascular disease and mental health problems.^2–4^ Access to wider clinical specialists, including general practitioners (GPs), is therefore becoming increasingly important so that people are cared for by staff with appropriate clinical skills.^5–8^

Access to healthcare in the UK, including treatment with HIV antiretroviral therapy, is universal and free at the point of delivery. In most instances, GPs are expected to be the first point of contact when access to any healthcare is required. Referrals to specialist secondary care facilities are then made if necessitated. However, unlike conditions, such as diabetes, GPs in many countries, including the UK, have not traditionally played a major role in PLWHIV's care in terms of managing either their infection or non-HIV-related issues.^5^ ^8^ ^9^ Their healthcare requirements have historically been provided by hospital-based secondary teams operating dedicated HIV outpatient clinics. While it is unclear whether UK GPs will have an increased role in managing PLWHIV's healthcare in the future, it is crucial that any changes, which are made to existing service arrangements, are evidence based and reflect people's needs and preferences.

The aim of this study was to understand which aspects of health services people diagnosed with HIV most value when seeking health advice, and hence which options they are most likely to use given a choice. More specifically, it starts with the premise that if the future objective is to increase GP involvement in the management of PLWHIV's health, then an understanding of their willingness to engage with GPs about new symptoms is an important step.

## Methods

We conducted a discrete choice experiment (DCE).^10^ ^11^ This is a cross-sectional questionnaire-based approach in which participants are required to choose between competing service options, in this study, an appointment with a GP or at an HIV clinic. The presented service options differed according to a number of ‘attributes’, such as waiting time for an appointment. Each attribute has a number of associated ‘levels’, such as ‘the same day’ (table 1), which vary by question. The underlying concept is that participants choose the option containing the combination of levels they most prefer. The full study protocol is available elsewhere.^12^

**Table 1:** Discrete choice attributes and levels

	Attribute	GP levels	HIV clinic levels
1.	The person you see is skilled at managing many general medical problems	Yes*	No*
2.	The person you see has the ability to refer you on to another healthcare professional if required	Yes*	Yes No†
3.	How quickly you will be seen	The same day	
		The next day	
		In 7 days	
		In 14 days†	
4.	An appointment outside of usual opening hours if you would like it	Unavailable†	Unavailable*
		Saturday 8:00–midday	
		Monday–Friday 17:00–20:00	
		8:00–20:00 7 days a week	
5.	How many times the healthcare professional has previously been seen	Never†	Never†
		Once in the last year	Once in the last year
		Twice in the last year	Twice in the last year
		More than twice in the last year	More than twice in the last year
6.	The type of person who is seen	A GP without specialist HIV training†	A consultant HIV doctor
		A GP with specialist HIV training	A doctor training to specialise in HIV
			An HIV specialist nurse
			An HIV specialist pharmacist†
7.	The level of information the healthcare professional has access to	All medical records, except HIV details†	Just the HIV medical records†
		All medical records, including HIV details	All medical records, including HIV details

*Indicates the levels on this attribute do not vary, meanings its impact in terms of choice is included in the relevant alternative specific constant term.

†Indicates base level for each attribute.

GP, general practitioner.

This DCE used a labelled approach, which is appropriate when the choices (a ‘GP’ or ‘HIV clinic’ appointment) are thought to be associated with important characteristics and feelings that are not specifically described by the attributes (ChoiceMetrics. *Ngene 1.1.1 User manual and reference guide*. 1.1.1 2014).^10^

### Choice of attributes and levels

The attributes and levels were derived from a systematic literature review^13^ and a qualitative study. The latter included people who were at least 16 years age and registered for care with an National Health Service (NHS) HIV clinic. A total of 74 people took part in 12 focus groups in Brighton and London, UK, between November 2013 and December 2014. Participants were quota sampled based on age (>50 yes/no), sex, sexual orientation (men who have sex with men (MSM)/heterosexual) and ethnicity (African/non-African). A topic guide, based on the literature review, was used to assess participant's experiences of existing HIV services and attitudes towards possible future developments. Data were analysed using a framework analysis approach.^14^ The final list of attributes and levels was determined by the study investigators over two face-to-face meetings (table 1). They were selected on the basis they represented current service practices or were seen as potentially realistic changes to them. The final draft list was reviewed by a GP with an interest in HIV medicine.

### Question framing

Before answering the questions, participants were asked to imagine they were currently receiving antiretroviral therapy and had been feeling well for the past 3 months. ‘Today’, however, they had developed one of the list of symptoms and had decided to seek medical advice for a headache, fever, rash, diarrhoea or abdominal pain. They were chosen on the basis of an audit of 50 of the most recent sets of notes for people who telephoned Brighton and Sussex University Hospitals NHS Trust's HIV outpatient triage service for advice about their health.

The initial DCE questionnaire was generated using an orthogonal approach. The final design used a Bayesian D-efficient approach basing priors on a pilot study consisting of 28 PLWHIV. The instrument was divided into two versions with each containing 12 DCE questions.

All attribute levels were dummy-coded (1 for group membership, 0 otherwise) except when estimating the alternative specific constant (ASC). This is the term that represents the extent to which people prefer a GP or HIV clinic appointment when all other factors are disregarded. Effects coding was used for ASC to avoid confounding with the attribute base levels on the main attributes. The parameters in all the DCE models were assumed to be alternative specific, meaning that the estimated ORs were specific to each service option (either a GP-clinic or HIV-clinic appointment) where appropriate.

The DCE responses were analysed using conditional logit (CLOGIT) and latent class models (LCM). The former is the basic form of analysis, but as the results represent responses for an average respondent, it may mask important heterogeneities. LCMs overcome this problem by grouping respondents into classes that have similar preferences and identifying characteristics associated with likely class membership, such as age (ChoiceMetrics. *Ngene 1.1.1 User manual and reference guide*. 2014).^15^ To identify these characteristics, and given that the subsequent LCM identified two classes based on inspection of the SEs and Akaike's information criteria,^16^ a series of univariate logit models were run using the following self-reported independent variables that were collected alongside the DCE responses: gender/sexuality (MSM, heterosexual male or female), HIV/sexuality disclosure to a GP (yes or no), year of diagnosis (before 1996, 1996–2000, 2001–2005, 2006–2010 or 2010+), clinic location (London, Brighton or other), ethnicity (white, black African, black other or mixed race/other), highest educational qualification (none, ‘O’ levels/GCSEs, ‘A’ levels, at least a degree or other), last CD4 <200 cells/mm^3^ (yes or no), nadir CD4 <50 cells/mm^3^ (yes or no), ‘perfect health’ recorded on the EQ-5D-3L^17^ health-related quality-of-life questionnaire (yes or no), full-time employment (yes or no) and number of current health problems (0, 2–4, 5–6 or 6–16). Variables that included at least one statistically significant category at the 5% level were entered into a single multivariable logistic regression using the likelihood of class 1 membership (>50% probability, yes or no) as the dependent variable. Independent variable category definitions were varied in a sensitivity analysis.

Data collection took place between December 2014 and April 2015 in 14 HIV clinics in London and across the Kent, Surrey and Sussex-Clinical Research Network. All participants were at least 16 years of age and had been diagnosed with HIV for a year or more. Participants attending general HIV clinics were asked to complete the questionnaire by research staff. However, in order to assess how representative participants' were of the clinics at which they were recruited, comparisons were made with a large UK-based cohort of PLWHIV known as UK CHIC.^18^

## Results

A total of 1106 questionnaires were returned; 97.6% of DCE responses were completed.

Thirty-eight per cent of respondents were aged 50 or over, most were men (85%), white (74%) and MSM (69%). Almost 50% had a CD4 count >500 cells/mm^3^ and 93% were receiving combination antiretroviral therapy (cART) (table 2). Over 95% were registered with a GP, 87% had disclosed their HIV status to their GP and 74% stated that their GP knew their sexuality.

**Table 2:** Respondent demographics

	All DCE respondents		Restricted DCE respondents*		CHIC
Characteristic	n	Median (IQR) or %	n	Median (IQR) or %	Median (IQR) or %
Age in years	1069	46.0 (38.0–52.0)	892	46.9 (38.0–52.0)	45.0 (38.0–51.0)†
>50 years	1069	38.0	892	37.0	–
EQ-5D-3L_utility_	952	0.85 (0.69–1.00)	742	0.85 (0.69–1.00)	–
Gender					
Male	922	85.3	793	87.6	81.6†
Female	156	14.4	110	13.3	18.4†
Transgender	3	0.3	2	0.2	–
Sexual preference‡					
Heterosexual	267	25.1	186	20.9	26.1†
Homosexual§	736	69.0	655	73.4	67.2†
Bisexual	43	4.0	33	3.7	–
Prefer not to say	20	1.9	18	2.0	–
Ethnicity					
White	794	74.0	669	74.8	64.6†
Black African	150	14.0	110	12.3	18.6
Black other	29	2.7	25	2.8	5.1
Other/mixed race	100	9.3	91	10.2	9.9
Clinic location					
London	584	52.8	–	–	–
Brighton	342	30.9	–	–	–
Other	180	16.3	–	–	–
Highest qualification					
None	100	9.5	–	–	–
GCSE/‘O’ levels	204	19.3	–	–	–
‘A’ levels	194	18.4	–	–	–
Degree or above	482	45.7	–	–	–
Other	75	7.1	–	–	–
In full-time employment					
Yes	585	55.3	–	–	–
No	473	44.7	–	–	–
Last CD4 count (cells/mm^3^)					
<200	107	10.1	80	10.2	2.5†
200–349	109	10.3	87	11.2	8.3†
350–500	195	18.4	156	20.1	20.6†
More than 500	525	49.4	455	58.5	68.7†
Unsure	126	11.9	–	–	–
Lowest CD4 count (cells/mm^3^)					
<50	212	20.3	174	24.1	12.4†
50–100	120	11.5	94	13.0	10.9†
101–200	175	16.8	152	21.0	24.9†
201–350	210	20.1	171	23.7	31.8†
>350	150	14.4	132	18.3	20.0†
Unsure	176	16.9	–	–	–
Year diagnosed					
Before 1996	197	18.9	–	–	–
1996–2000	240	15.7	–	–	–
2001–2005	242	23.3	–	–	–
2006–2010	163	23.1	–	–	–
After 2010	198	19.0	–	–	–
Currently receiving cART¶	1003	92.7	838	92.3	88.9
Current health problems					
None	317	32.9	–	–	–
1–2	387	40.1	–	–	–
3–4	158	16.4	–	–	–
5–6	66	6.8	–	–	–
7–16	37	3.8	–	–	–
Registered with a GP					
Yes	1031	95.7	–	–	–
No	46	4.3	–	–	–
Does GP know your HIV status¶					
Yes	906	86.5	–	–	–
No	142	13.5	–	–	–
Does GP know your sexuality**					
Yes	765	73.6	–	–	–
No	274	26.4	–	–	–

Some numbers do not sum exactly to 1106 or 100% due to missing values and/or rounding.

*London and Brighton respondents only.

†UK CHIC (*n*=14 972).

‡For UK CHIC data this was defined as mode of HIV acquisition.

§All were either male or transgender.

¶Responses indicating a person was unsure if a GP knew their HIV status were coded as the GP ‘not knowing’.

**MSM or bisexual participants who indicated that they were unsure if their GP knew their sexuality were coded as the GP ‘not knowing’, whereas a GP was indicated as ‘knowing’ if a person was heterosexual.

DCE, discrete choice experiment; GP, general practitioner; MSM, men who have sex with men.

Five of the participating centres (*n*=926/1106, 84%) were also in UK CHIC (*n*=14 972). Comparisons showed that the samples were similar in terms of age and the proportion of people receiving cART (table 2). However, UK CHIC contained proportionately fewer MSM, white people, and participants were less likely to be in the poorer nadir/current CD4 categories.

### Conditional logit model

The basic model correctly predicted almost 68% of responses. The ASC indicated people were more likely on average to choose a GP rather than an HIV clinic appointment when all other factors are disregarded (OR 1.17, 95% CI 1.13 to 1.22).

#### Attributes common to both clinic service options

**Figure 1:**
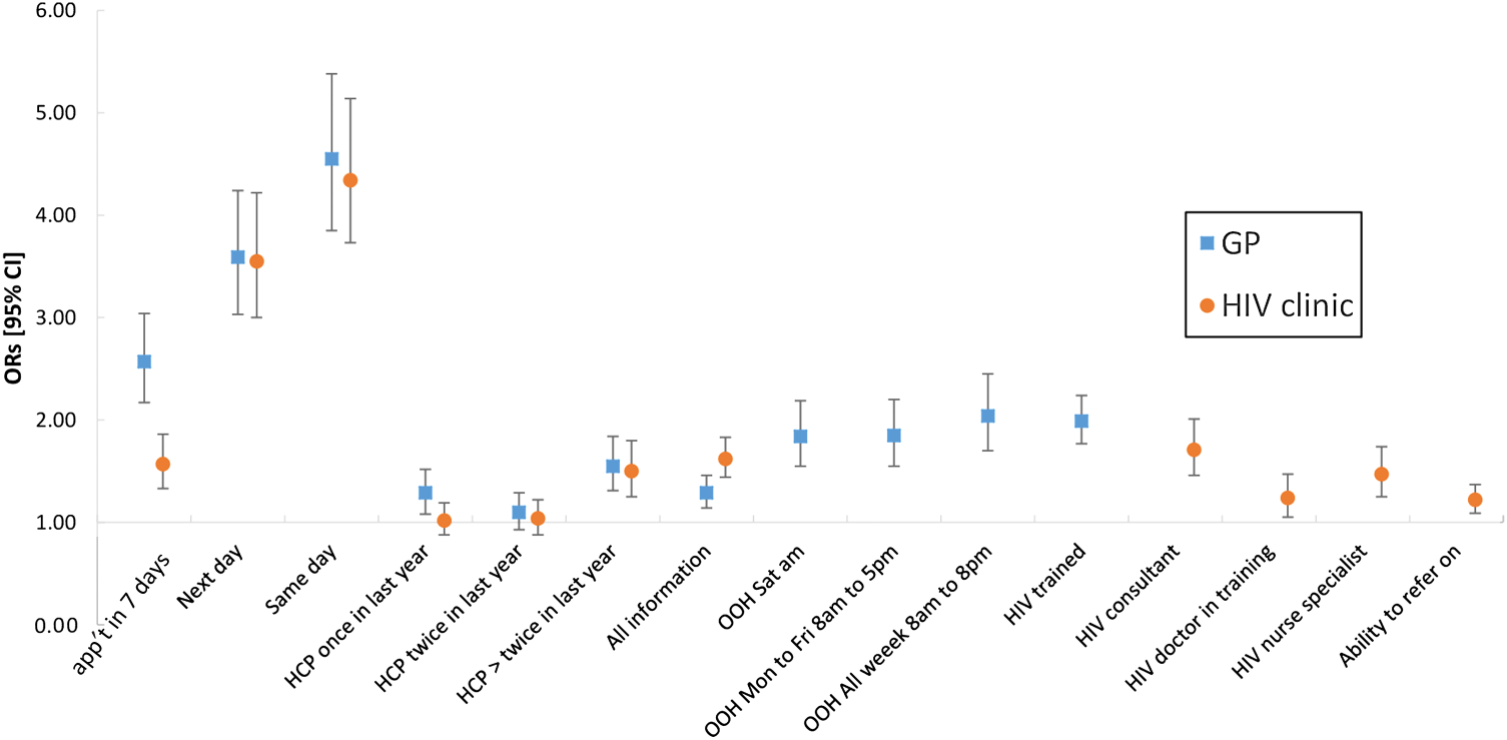
Conditional logit model results. App't, appointment; GP, general practitioner; OOH, out-of-hours.

The ORs from the CLOGIT model for each attribute level are shown in figure 1. They show respondents particularly valued shorter waiting times for appointments (ORs between 1.52 and 3.62, p<0.001 in all instances). It also showed that people had strong preferences for appointments with either GPs (OR 1.46, 95% CI 1.29 to 1.65) or HIV healthcare professionals (HCPs) (OR 1.32, 95% CI 1.16 to 1.51) who they had seen at least three times in the previous year rather than not at all. However, the evidence that participants preferred appointments with HCPs who they had seen once or twice in the last year, rather than not at all, was generally weak. Respondents preferred, on average, for GPs (OR 1.29, 95% CI 1.18 to 1.42) and HIV clinic staff (OR 1.57, 95% CI 1.44 to 1.72) to have access to all their medical records, rather than partial information.

#### Attributes that differed by clinic service options

Participants valued out-of-hours (OOH) GP services, with ORs between 1.64 and 1.85 (p<0.001 in all instances) depending on opening times compared with appointments within normal working hours only. Respondents strongly valued appointments with GPs who had specialist HIV training compared with those without it (OR 1.86, 95% CI 1.71 to 2.03). The ability of HIV professionals to refer people on to specialist doctors if required was valued by participants compared with referral back to a GP (OR 1.22, 95% CI 1.13 to 1.33).

### Latent class model

The LCM increased the proportion of correctly predicted choices to 84% and was a statistically better fit to the data. Two classes were identified, with 59% and 41% of participants likely to be in classes 1 and 2, respectively.

### Class preferences

**Figure 2:**
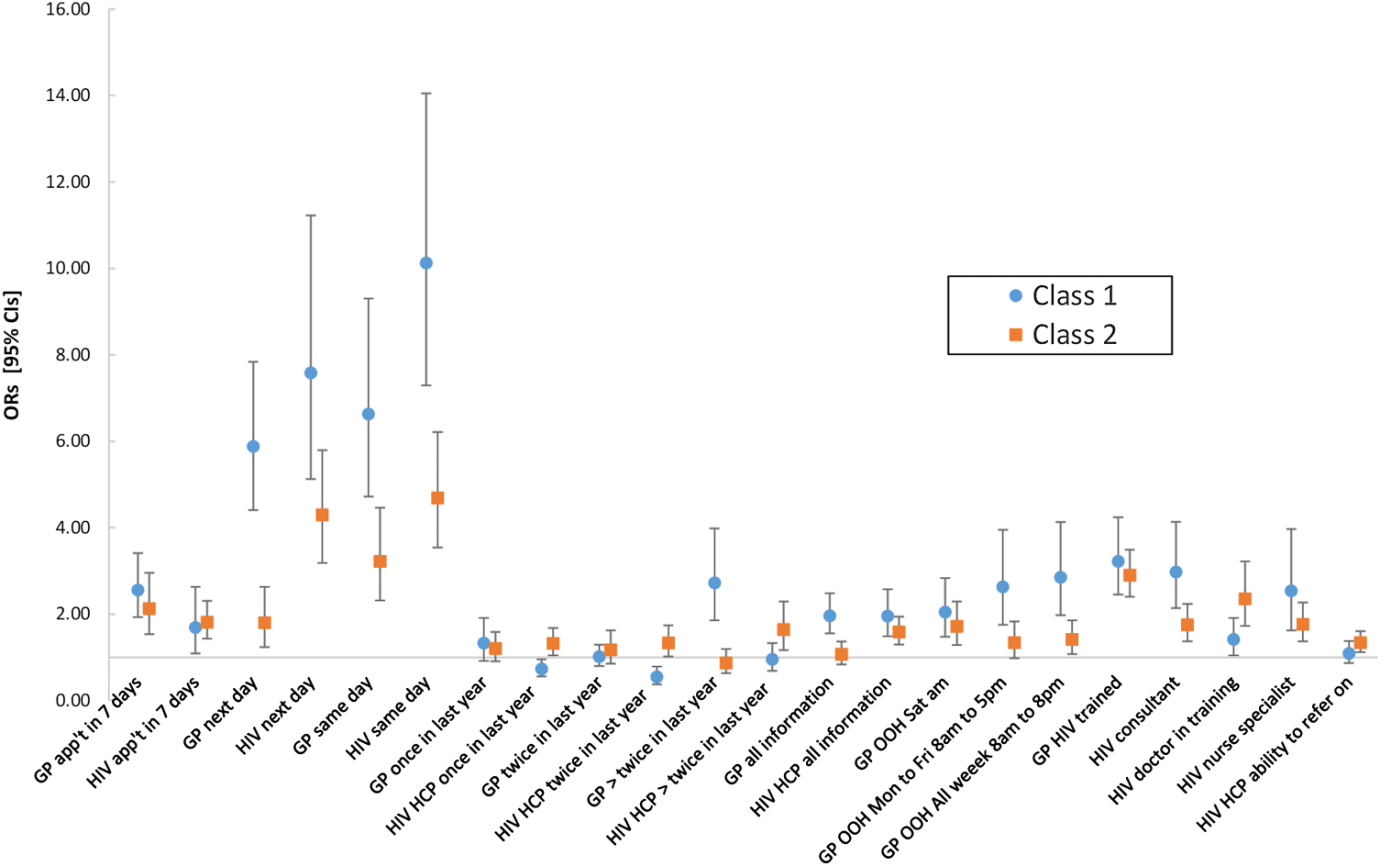
Latent class model results. App't, appointment; GP, general practitioner; OOH, out-of-hours.

The ASC indicated that people who were likely to be in class 1 preferred GP to HIV clinic appointments, when all other factors are disregarded (OR 4.39, 95% CI 3.82 to 5.10) and appeared to particularly value timely appointments. For example, figure 2 shows the ORs associated with ‘the same’ or ‘next day’ waiting times, compared with in ‘2 weeks’, were all high irrespective of service option (ORs ≥5.88 and p<0.001 in all instances). Moreover, they also valued OOH GP services compared with appointments during ‘normal working hours only’ (ORs ≥2.05 and p<0.001 in all instances) and were less concerned about how many times they had previously seen an HIV HCP or if they had seen their GP twice or less in the previous year (p>0.05 in both instances). However, they strongly preferred appointments with GPs who they had seen more than twice in the last year (OR 2.72, 95% CI 1.86 to 3.99) and who had received specialist HIV training (OR 3.22, 95% CI 2.45 to 4.24). For HIV clinic appointments, people more likely to be in class 1 most favoured seeing an HIV consultant of all the HCP options (OR 2.98, 95% CI 2.14 to 4.14). They also preferred appointments where GPs (OR 1.96, 95% CI 1.55 to 2.48) and HIV HCPs (OR 1.96, 95% CI 1.49 to 2.57) had all their clinical information available.

People who were more likely to be in class 2 differed in that they placed a particularly high value on having HIV clinic rather than GP appointments, when all other factors were disregarded (OR 3.63, 95% CI 3.20 to 4.11). They also preferred shorter to longer appointment waiting times (ORs ≥1.80 and p≤0.002 in all instances); however, compared with class 1 members, most of the associated ORs were noticeably lower, suggesting waiting times were generally of lesser importance to them, as were GP OOH appointments. Class 2 members appeared to be indifferent as to how many times they had seen their GP over the past year (all associated p values >0.05). However, they valued having seen their HIV HCP at least once in the last year compared with not at all (ORs >1 and p<0.05 in both instances). Although their overall strength of preference for GP appointments was low, class 2 members revealed a strong preference for appointments with GPs who had received specialist HIV training rather than none (OR 2.90, 95% CI 2.41 to 3.49). Moreover, they valued their HIV HCP having access to all their clinical information compared with partial information only (OR 1.59, 95% CI 1.30 to 1.95), but not their GP (OR 1.07, 95% CI 0.84 to 1.36).

### Class predictors

Multivariable logit analysis showed that people in perfect health (OR 1.56, 95% CI 1.16 to 2.11) and those who had disclosed their HIV status to their GP (OR 2.31, 95% CI 1.45 to 3.67) were more likely to be in class 1 than class 2. None of the remaining variables remained predictive of clinic choice in the multivariable model. Varying the category definitions had negligible impact on the results.

## Discussion

This study suggests people diagnosed with HIV for at least a year who require medical advice for new symptoms value a number of service characteristics, particularly quick appointments. However, respondents were divided in terms of how they generally valued GP services.

A London-based study by Weatherburn *et al*^9^ (*n*=1390) found that about a third of people who had disclosed their HIV infection to their GP could not think of ways in which GP services could be improved. However, the remaining two-thirds stated the importance of longer opening hours, shorter waiting times and improved appointment booking systems. They also wanted their GPs to become more knowledgeable about HIV. Our results generally support these findings but increase our understanding of the trade-offs that people with HIV consider when accessing primary or secondary care facilities. Weatherburn *et al* also conclude that many people are highly satisfied with the current UK model of care but that many people are open to GPs having more involvement in their care. Again, our results broadly support this conclusion in so much that there appears to be a clear split between people who value GP appointments and those who do not. Hutchinson *et al*^8^ suggest that those who are most likely to view primary care as an alternative have disclosed their HIV status. Our LCM analysis results support this finding but also suggests that the same is true for people who are in excellent self-reported health.

The LCM results showed that while people who were likely to be in class 1 indicated a preference for GPs to have access to all information. Those more likely to be in class 2 were indifferent between this and only having access to their non-HIV records. The results in themselves do not indicate why, but concerns about levels of confidentiality with wider clinical specialities, including GPs, is a known concern to PLWHIV's.^19^ Indeed, for this reason in the UK at least, recording systems in sexual health and HIV are separate from all other NHS organisations. These results suggest that the issue of sharing HIV-positive people's medical records is complex because while the clinical importance of linking records is likely to increase, people's views on it are divided.

The major strengths of this preference study are its large sample size and its discrete choice design; it requires participants to make choices by ‘trading off’ different service characteristics thus is considered to be more realistic than simply asking people their preferences. However, there are a number of limitations with it. First, while the question framing, attributes and levels were chosen to be as realistic as possible it is a hypothetical exercise. Second, compared with data collected at corresponding centres available via UK CHIC, our study participants were more likely to be white, MSM and to be in the poorer nadir/current self-reported CD4 strata; it is difficult to know how these differences might have impacted the results. However, none of these variables were predictive of class membership in the LCM analysis. Third, a number of attributes raised in the qualitative analysis were excluded from the DCE design, either because of a need to limit the number of questions or because they were difficult to operationalise. For example, the need to ‘trust’ a GP was frequently raised yet its meaning varied by participant. While a number of attributes were included to encapsulate this factor, such as frequency of HCP contact over the previous year and the amount of information they have access to, we acknowledge that they do not include all issues of concern such as staff using appropriate language.^19^ Fourth, we chose not to recruit people who had been diagnosed with HIV within the last year, on the basis that they would probably be advised to attend their usual HIV clinic if they developed a new health symptom. However, if the aim is to encourage people with HIV to use GP services more frequently in the future, then perhaps those who are newly diagnosed are an important group to consider. Last, the question framing included a list of symptoms as a ‘prompt’ for seeking health advice. However, the pilot study indicated that for more general symptoms, participants were much more willing to see GPs suggesting PLWHIVs preferences for using HIV clinic or GP services are likely to be sensitive to presenting symptoms.

Hutchinson *et al*^8^ recently stated that increased GP involvement in caring for people with HIV could have potential benefits, as they have expertise in managing non-microbial HIV-associated comorbidities such as mental health issues and cardiovascular disease, particularly for people with stable infection. Our study suggests that many people with stable infection would be willing to try shared care arrangements with GPs, particularly those who are already registered with a GP, and with GPs who have specialist HIV knowledge. However, we agree with Hutchinson that further research is required to establish clinical and economic outcomes of specific shared care arrangements before they can be recommended more formally as the optimal service model.

**Key messages**

- People living with HIV (PLWHIV) value many aspects of care when seeking advice for new health problems, but shorter waiting times are particularly important.
- PLWHIV were divided in their willingness to engage with general practitioners (GPs). Sixty per cent of respondents indicated that they valued GP appointments independently of the described service characteristics.
- However, responses from the remaining 40% showed a strong general unwillingness to engage with GPs even though there were some perceived advantages.
